# Preliminary neutron diffraction analysis of challenging human manganese superoxide dismutase crystals

**DOI:** 10.1107/S2053230X17003508

**Published:** 2017-03-29

**Authors:** Jahaun Azadmanesh, Scott R. Trickel, Kevin L. Weiss, Leighton Coates, Gloria E. O. Borgstahl

**Affiliations:** a Eppley Institute for Research in Cancer and Allied Diseases, 987696 Nebraska Medical Center, Omaha, NE 68198-7696, USA; b Department of Biochemistry and Molecular Biology, 985870 Nebraska Medical Center, Omaha, NE 68198-5870, USA; c Biology and Soft Matter Division, Oak Ridge National Laboratory, 1 Bethel Valley Road, Oak Ridge, TN 37831, USA

**Keywords:** manganese superoxide dismutase, neutron diffraction, perdeuteration, human, large unit cell

## Abstract

Superoxide dismutases (SODs) are enzymes that protect against oxidative stress by dismutation of superoxide into oxygen and hydrogen peroxide through cyclic reduction and oxidation of the active-site metal. The complete enzymatic mechanisms of SODs are unknown since data on the positions of hydrogen are limited. Here, methods are presented for large crystal growth and neutron data collection of human manganese SOD (MnSOD) using perdeuteration and the MaNDi beamline at Oak Ridge National Laboratory. The crystal from which the human MnSOD data set was obtained is the crystal with the largest unit-cell edge (240 Å) from which data have been collected *via* neutron diffraction to sufficient resolution (2.30 Å) where hydrogen positions can be observed.

## Abstract 2

The growth of crystals of perdeuterated human manganese superoxide dismutase with a unit-cell edge of 240 Å was optimized for neutron data collection. Data were collected to 2.30 Å resolution from a 0.26 mm^3^ crystal. This is the crystal with the largest unit-cell edge from which data have been collected *via* neutron diffraction to sufficient resolution where hydrogen positions can be observed.

## 1. Introduction   

Superoxide dismutases (SODs) are necessary antioxidant enzymes that form the first line of defense against oxidative stress by converting the reactive oxygen species superoxide into oxygen and hydrogen peroxide. Remediation of superoxide is performed by cyclic dismutation of the active-site metal, where both the oxidized and reduced forms of the metal contribute to catalysis (Holm *et al.*, 1996 ▸). 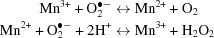
In eukaryotes, MnSOD is found in the mitochondrial matrix and protects against the superoxide generated by electron leakage from the electron-transport chain (Jastroch *et al.*, 2010 ▸). Mice with MnSOD knockout die within the first day of life, and MnSOD mutations are associated with numerous diseases (Li *et al.*, 1995 ▸; Kim, 2010 ▸; Perry *et al.*, 2007 ▸).

Despite their biological importance, the complete multistep enzymatic mechanisms of SODs are unknown owing to limitations in identifying the hydrogen positions at the active site. Knowledge of the hydrogen positions is critical because (i) they are needed to differentiate the ligands in the active site, which are hypothesized to differ based on the redox state of the active-site metal (Lah *et al.*, 1995 ▸), and (ii) they reveal the source and the pathway of protons to the catalytic center for proton-assisted electron transfer.

All X-ray crystal structures of MnSOD and FeSOD have an O atom coordinated to the active-site metal which is thought to be involved in proton-assisted electron transfer. Whether the oxygen is part of a water molecule or a hydroxide ion is not known owing to the low X-ray scattering factor of H atoms and insufficient diffraction resolution. The hydrogen content of the molecule has been postulated to depend on the redox state of the metal (Abreu *et al.*, 2005 ▸; Miller *et al.*, 2003 ▸). Discerning between superoxide and hydrogen peroxide is also difficult in X-ray crystallography. Hydrogen peroxide is produced during the oxidation step of the metal and dissociation of the oxidized product-inhibited complex. X-ray crystal structures of fully oxidized metalloproteins cannot be measured since photoelectrons reduce the metals during X-ray data collection (Carugo & Djinovic Carugo, 2005 ▸). This is an advantage of neutron crystallography, where the oxidation state of the active-site metal is unaffected by the beam.

The source and pathway of the proton transfer(s) used for the dismutation of superoxide is not known. A conserved hydrogen-bond network involving the O atom coordinated to the metal and several residues has been presumed to shuttle protons from bulk solvent to the catalytic center (Abreu & Cabelli, 2010 ▸; Ramilo *et al.*, 1999 ▸; Lah *et al.*, 1995 ▸). Mutation of the residues contributing to the hydrogen-bond network has been shown to highly affect the catalytic activity and has suggested two differing pathways for the reduction of superoxide (Abreu & Cabelli, 2010 ▸; Greenleaf *et al.*, 2004 ▸; Lévêque *et al.*, 2000 ▸; Hearn *et al.*, 2003 ▸).

Neutron diffraction of human MnSOD is being used to uncover the hydrogen positions, the effect of the metal oxidation state, the proton-relay pathway and the catalytic mechanism of SODs. Here, methods are presented for the production of perdeuterated human MnSOD, purification, large crystal growth and neutron data collection to 2.30 Å resolution using the MaNDi single-crystal diffractometer at Oak Ridge National Laboratory (ORNL). Perdeuteration, replacing every H atom with deuterium, was performed using the Biodeuteration Laboratory at ORNL. Perdeuteration is advantageous as it decreases the incoherent scattering by 40-fold compared with a hydrogenated sample, thereby reducing the background and increasing the resolution of diffraction. Our crystal system was particularly challenging for neutron diffraction owing to the large unit-cell edge of 240 Å. The crystal used to obtain the human MnSOD data set is the crystal with the largest unit-cell edge from which data have been collected *via* neutron diffraction to sufficient resolution where hydrogen positions can be observed.

## 2. Materials and methods   

### 2.1. Adaptation to deuterium   

Full-length human MnSOD cDNA optimized for *Escherichia coli* codons was cloned into the pCOLADuet-1 expression vector and transformed into BL21(DE3). Cells were grown in Terrific Broth with 0.8%(*v*/*v*) glycerol while cell strain fidelity was maintained with 30 µg ml^−1^ of kanamycin for initial growth and deuterium adaptation. Deuterium adaptation was performed by subculturing from Terrific Broth to H_2_O minimal media and subsequently subcultured into minimal media containing increasing D_2_O content (25, 50, 75 and 100%). Subculturing was performed by 1:5 dilutions into 3 ml of media from an OD_600_ of 1 to a final of 0.2. The composition of minimal medium was as follows: 5 g l^−1^ glycerol, 7 g l^−1^ (NH_4_)_2_SO_4_, 0.5 g l^−1^ ammonium citrate dibasic, 5.25 g l^−1^ Na_2_HPO_4_, 1.6 g l^−1^ KH_2_PO_4_, 1 ml l^−1^ of 20% MgSO_4_·7H_2_O, and 1 ml l^−1^ of trace elements solution (Larsson & Tornkvist, 1996 ▸). The 1000× trace elements solution contained 0.500 g l^−1^ CaCl_2_·2H_2_O, 0.098 g l^−1^ CoCl_2_, 0.160 g l^−1^ CuSO_4_·5H_2_O, 16.7 g l^−1^ FeCl_3_·6H_2_O, 0.114 g l^−1^ MnSO_4_·H_2_O, 22.3 g l^−1^ Na_2_EDTA·2H_2_O, and 0.180 g l^−1^ ZnSO_4_·7H_2_O (Holme *et al.*, 1970 ▸). Once adapted to D_2_O, glycerol stocks were stored at −80°C for future use.

### 2.2. Perdeuterated expression   

In preparation for perdeuterated expression, 25 µl of glycerol stock was used to seed 3 ml of D_2_O minimal medium, which was subsequently shaken at 250 rev min^−1^ and 37°C. Once the culture reached an OD_600_ of ∼1, it was subcultured 3× in D_2_O minimal medium at 1:20 dilution. From this point forward, the D_2_O minimal media contained 100 µg ml^−1^ kanamycin, 5 g l^−1^ D_8_-glycerol, and minimal medium salts that had been dried and exchanged three times using a rotary evaporator prior to dissolution in D_2_O (Meilleur *et al.*, 2009 ▸). All other solutions were prepared with D_2_O and filter sterilized into dry, sterile containers prior to use. Prior to inoculation, a 2.5 l bioreactor vessel was steam-sterilized and dried using sterile-filtered compressed air that had been first passed through a gas purifier containing Drierite^®^ and 5 Å molecular sieves. Upon dryness, fresh perdeuterated minimal medium (1.4 l) was added to the vessel. Sensors for monitoring pH and dissolved oxygen were rinsed with deionized water and 70% ethanol prior to installation through the vessel headplate. Temperature and compressed air flow were set at 30°C and 1.5 l min^−1^ (1 vvm) respectively. To inoculate the vessel, 50 ml of D_2_O-adapted cells were diluted to an initial OD_600_ of 0.14. Throughout the experiment, agitation was increased from 200 rev min^−1^ to maintain dissolved oxygen above a set point of 30% and 10%(*w*/*w*) NaOD was added on demand to control pD (>7.3). Upon depletion of D_8_-glycerol, the dissolved oxygen spike was used to initiate addition (9 ml h^−1^) of a feed solution consisting of 10%(*w*/*v*) D_8_-glycerol and 0.2% MgSO_4_ in 99.8% D_2_O. Upon reaching an OD_600_ = 8.3 after 21.8 h, a D_2_O-exchanged solution of MnSO_4_ was added to 1.4 g l^−1^and perdeuterated human MnSOD overexpression was induced by adding IPTG to a final concentration of 1 m*M*. After the 26.2 h induction, the cell suspension was collected and pelleted at 6000*g* *via* centrifugation at 4°C for 30 min to yield 63.7 g wet weight of perdeuterated cell paste.

### 2.3. Purification   

For purification, an approximately 15 g aliquot of cell pellet were resuspended in 50 m*M* potassium phosphate pH 7.8, adjusted by altering the ratio of dibasic (K_2_HPO_4_) and monobasic forms (KH_2_PO_4_) to 91 and 9%, respectively (Cold Spring Harbor Laboratory, 2006 ▸). Lysis was conducted with an Emulsiflex-C3 and the clarified lysate was incubated at 60°C for 1 h to precipitate contaminant proteins, which were removed by centrifugation. Soluble protein was dialyzed against 5 m*M* potassium phosphate pH 7.8 and applied to pre-swollen diethylaminoethyl (DE52) cellulose resin (GE Healthcare). The protein–resin slurry was rocked for 1 h at 10°C and poured into a Büchner funnel with Whatman No. 4 filter paper while under vacuum. Once dry, the resin was washed with 5 m*M* potassium phosphate pH 7.8 and the protein was then eluted with 100 m*M* potassium phosphate pH 7.8. The eluted protein was dialyzed against 2.5 m*M* 2-(*N*-morpholino)ethanesulfonic acid (MES) pH 5.5, applied onto a carboxymethyl (CM) Sepharose Fast Flow (GE Healthcare) column, eluted with a sodium chloride gradient and concentrated to 21 mg ml^−1^ using 5 kDa molecular-weight cutoff concentrators. The concentration was measured using a NanoDrop ND-1000 spectrophotometer using an extinction coefficient of 40 500 *M* ^−1^ cm^−1^ at 280 nm (Lévêque *et al.*, 2001 ▸). The purifications of hydrogenated and deuterated protein were identical.

### 2.4. Crystallization   

Optimization of the growth of large, hydrogenated crystals was conducted first. A protocol for hanging-drop vapor-diffusion crystal growth was translated in exactly the same concentrations to nine-well glass-plate sandwich-box setups (sitting-drop vapor diffusion; Hampton Research) and scaled up to 100 µl drops with differing ratios of protein to well solution at 21 mg ml^−1^ to screen for large crystal growth. The well solution consisted of 1.8 *M* potassium phosphate pH 7.8. Crystal setups were placed on neoprene vibration-isolation pads (Grainger 5XR47) and kept inside an incubator at 20°C (RUMED Rubarth Apparate GmbH). Ratios of protein to well solution of 3:2, 1:1 and 2:3 yielded the largest and highest quality crystals, as measured by X-ray diffraction resolution and mosaicity. The growth of large crystals was also achieved at 10°C using a higher salt concentration of 2.5 *M* potassium phosphate pH 7.8. Crystals grew to 3 mm^3^ after a month, although the largest crystals all had imperfections and/or diffracted X-rays poorly.

To identify whether differing crystallization conditions were needed for perdeuterated crystals, a hanging-drop grid screen was performed using 2 µl drops of 1:1 protein to well solution with protein at 21 mg ml^−1^ and with varying pH and concentrations of potassium phosphate both at room temperature and 10°C. Growth utilizing a reservoir solution consisting of 1.93 *M* potassium phosphate pH 7.8 at room temperature and 2.50 *M* potassium phosphate pH 8.0 (adjusted by altering the ratios of dibasic and monobasic forms to 94 and 6%, respectively) at 10°C consistently yielded single-crystal growth. The condition was translated to perdeuterated large crystal growth using 100 µl drops with protein (at 21 mg ml^−1^) to well solution ratios of 3:2, 1:1 or 2:3. Large-volume crystals were grown at 20°C in the same glass trays and incubator setup as described above. In these setups, perdeuterated crystals grew to 2 mm^3^ after three weeks, although the largest crystals diffracted X-rays poorly and displayed high mosaicity. Crystals from room temperature and 10°C were analysed. A 0.26 mm^3^ perdeuterated crystal grown in the room-temperature setup ranked the best and was used to collect our neutron data set (Fig. 3).

### 2.5. Solutions used for vapor exchange with deuterium   

To vapor-exchange titratable H atoms in the mounted crystals with deuterium, a suitable deuterated substitute reservoir solution was needed. For crystals grown in 1.93 *M* potassium phosphate pH 7.8 at 20°C, a total substitution of reservoir with 2.43 *M* deuterated potassium phosphate pH 7.4 (pD 7.8) maintained crystal stability. Likewise, crystals grown in 2.50 *M* potassium phosphate pH 8.0 at 10°C maintained stability on substitution with 3.00 *M* deuterated potassium phosphate pH 7.6 (pD 8.0). In general, a deuterated substitute reservoir solution 0.5 *M* above our native reservoir concentration caused no observable alterations in our crystals.

### 2.6. Preparation for neutron diffraction   

To prepare for neutron data collection, crystals were mounted in quartz capillaries, vapor-exchanged with deuterium, screened for diffraction viability using X-rays and safely transported by airplane from the home laboratory to ORNL. These steps are described in detail below.

#### 2.6.1. Crystal mounting   

For mounting crystals, thick-walled quartz capillaries (1.5 mm inner diameter × 1.8 mm outer diameter; VitroCom catalog No. CV1518-Q-100) were preferred owing to their transparency to neutrons and their durability during handling and travel. Crystals were carefully drawn into the capillaries from their crystallization drops using a Captrol III aspirator (Drummond Scientific) connected to rubber tubing with vacuum grease. Approximately 20 µl reservoir solution was added to the crystallization drops prior to mounting to account for the solution being drawn into the capillary. Crystals fixed to the well plates could be dislodged by repeatedly pipetting around the crystal slowly using a standard pipette. Once crystals had been drawn into the capillaries, the native mother liquor was removed and the crystals were carefully dried using paper wicks to avoid crystal slippage. A conservative amount of mother liquor was left in a small pool with the crystal to prevent its drying. Slugs of deuterated substitute reservoir solution (10 µl) were placed on both sides of the crystal, and the capillary was sealed by heating and cooling beeswax. To ensure the saturation of deuterium exchange, capillaries were opened after one week with a heated syringe needle and the slugs of deuterated reservoir were replaced with fresh solution before being resealed.

#### 2.6.2. Screening for diffraction viability   

To preliminarily assess whether our crystals would be viable for neutron diffraction, diffraction was tested with a 5 s exposure to X-rays using the Omaha facility, which houses a Rigaku FR-E rotating-anode generator with VariMax HR optics and an R-AXIS IV^++^ detector. Exposures were kept as short as possible to avoid radiation damage, but had to be extended to 5 s owing to the thickness of the quartz capillary and the size of the crystal.

#### 2.6.3. Transport   

To further secure the beeswax seals of the capillaries, a thin layer of fingernail polish was applied prior to travel. Capillaries were either placed in 15 ml conical tubes surrounded by cotton wool or fastened in DVD cases with clay. Tubes and DVD cases were placed in cushioned areas of luggage during travel, such as between foam padding in a dedicated suitcase that was checked in or in a hand-carried backpack.

### 2.7. Data collection   

Upon arrival at ORNL and prior to data collection, the crystals were dried again to a minimal amount of mother liquor to minimize background signal and to avoid crystal slippage. Slugs of deuterated substitute reservoir solution were again placed on both sides of the crystal. Time-of-flight (TOF) neutron diffraction data from a perdeuterated crystal of 0.26 mm^3^ in volume at 293 K were recorded to 2.30 Å resolution using the MaNDi instrument (Coates *et al.*, 2010 ▸, 2015 ▸) at the Spallation Neutron Source (SNS) onsite at ORNL using all neutrons between 2 and 4 Å wavelength. The ω angle was fixed at 90° for data collection. The ω drive on MaNDi rotates the crystal in a horizontal circle parallel to the neutron beam. The crystal was held static for each image and was rotated by 20° on φ between images. Six images were collected in the beam time assigned for this project. These six images were processed and integrated using the *Mantid* package (Arnold *et al.*, 2014 ▸) and *LAUENORM* from the *LAUEGEN* package (Campbell *et al.*, 1998 ▸). *LAUENORM* performs a wavelength normalization of the Laue data and scaling between Laue diffraction images. X-ray diffraction data were collected from the same crystal at 293 K using an in-house rotating-anode Rigaku MicroMax-007 HF equipped with an R-AXIS IV^++^ detector and were processed using the *XDS* package (Kabsch, 2010 ▸) and *SCALA* from the *CCP*4 suite (Winn *et al.*, 2011 ▸). Owing to the large unit cell and several limitations of the in-house X-ray diffractometer at ORNL, which included a fixed 2θ angle and a minimum φ rotation, data-collection resolution was limited to 2.35 Å in order to maintain good spot separation (Table 1 ▸).

## 3. Results and discussion   

**Figure 1:**
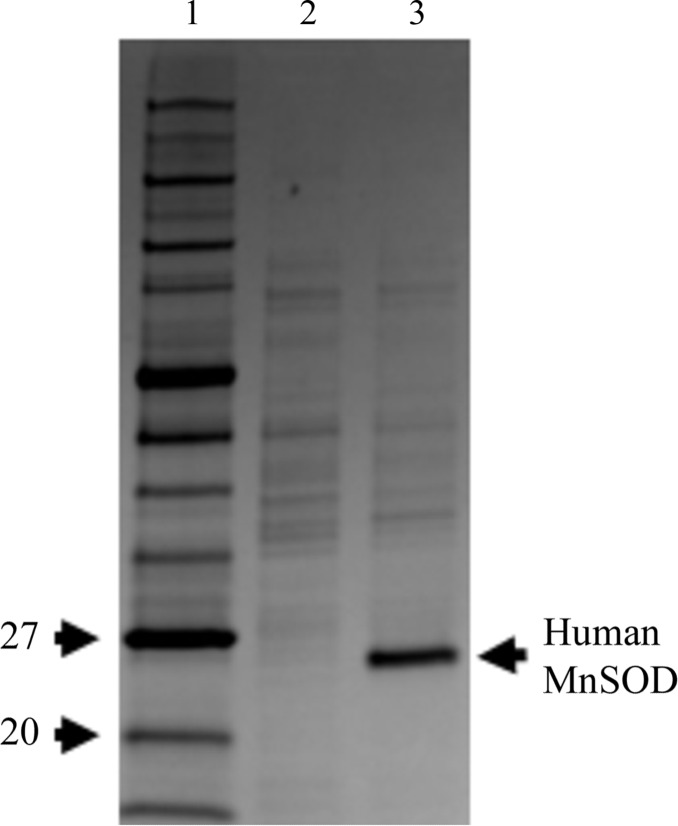
Fermentor growth of perdeuterated human MnSOD. SDS–PAGE of whole-cell lysate from cells grown in perdeuterated media immediately before and 13 h after induction. Monomeric human MnSOD has a molecular weight of 22 kDa. Samples were normalized to equivalent optical densities. Lane 1, molecular-mass markers (labelled in kDa). Lane 2, pre-induction. Lane 3, post-induction.

**Figure 2:**
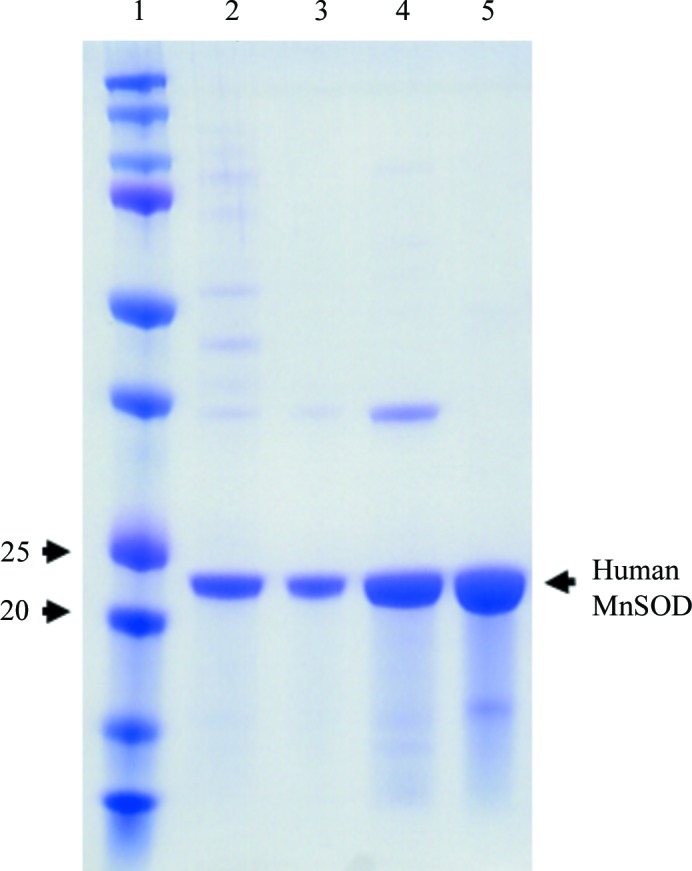
Purification of human MnSOD. SDS–PAGE of perdeuterated human MnSOD during purification. Purification steps are sequential from left to right. Lane 1, molecular-mass markers (labelled in kDa). Lane 2, whole-cell lysate. Lane 3, post-heat (60°C). Lane 4, post-anion exchange. Lane 5, post-cation exchange. Heat was applied by placing samples in a water bath, anion exchange was conducted using DE52 resin and eluting with 100 m*M* potassium phosphate pH 7.8, and cation exchange was performed using a CM column and eluting with a sodium chloride gradient. See §[Sec sec2]2 for further details of purification.

To produce deuterated human MnSOD, *E. coli* cells harboring the recombinant plasmid needed to be grown in deuterated minimal medium. The *sodA^−^sodB^−^* strain of *E. coli*, which lacks endogenous MnSODs and FeSODs (Steinman, 1992 ▸) and was used for our experiments with hydrogenated proteins, failed to grow in minimal medium. BL21(DE3) cells with the endogenous SODs were subsequently used and could grow and adapt to fully deuterated medium. Adaptation was performed by subculturing from Terrific Broth to H_2_O minimal medium and subsequent subcultures into increasing ratios of deuterium-labelled minimal medium (25, 50, 75 and 100%) at an OD_600_ of 1. Subculturing was performed by 1:5 dilutions into 3 ml medium from an OD_600_ of 1 to a final OD_600_ of 0.2. The expression of recombination human protein upon induction with IPTG was verified by SDS–PAGE (Fig. 1 ▸). The purification of human MnSOD was performed using nearly identical protocols as used for the hydrogenated form (Fig. 2 ▸). Contamination by dimeric *E. coli* SODs was not detected on native gels (data not shown). Any concerns about such contamination were relieved when the perdeuterated sample crystallized in a space group and unit cell that are only associated with human MnSOD.

**Figure 3:**
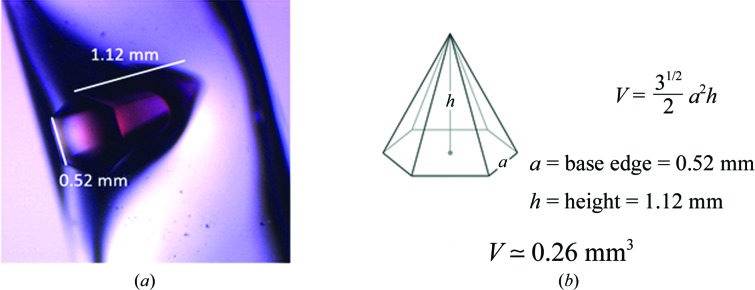
Crystals of perdeuterated human MnSOD. (*a*) A 0.26 mm^3^ hexagonal pyramidal crystal of perdeuterated human MnSOD mounted in a quartz capillary for neutron data collection. Measurements of base edge and height were used for volume calculation as in (*b*).

Prior to obtaining our neutron data set from a perdeuterated crystal, we attempted to collect data from hydrogenated crystals of 1.0–1.2 mm^3^ in size with the titratable H atoms exchanged with deuterium. Although these crystals diffracted well using X-rays, neutron data collection only yielded diffraction to 2.95 Å resolution, a resolution that is insufficient to accurately observe hydrogen positions. The extent of diffraction was attributed to the large unit-cell edge of 240 Å and incoherent scattering by the H atoms at non-titratable positions. To improve the diffraction intensities and extend the diffraction limit, data were later collected from perdeuterated crystals (Table 1 ▸). The highest diffraction limit, 2.30 Å, was achieved with a 0.26 mm^3^ perdeuterated crystal (Fig. 3 ▸). Reasonable completeness for neutron diffraction was achieved with only six images owing to the high symmetry of the space group.

**Figure 4:**
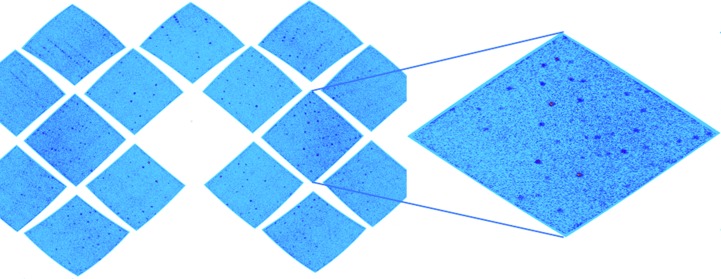
The diffraction pattern of human MnSOD from the spherical detector orientation of MaNDi. A selected time-of-flight slice of neutrons with wavelength of 3.0–3.1 Å is shown. A close-up view of a single detector module is included.

Large unit-cell axes are highly problematic in Laue diffraction experiments owing to the large number of closely packed reflections that they generate (Blakeley, 2011 ▸). Without the use of TOF methods, which enable a multiwavelength Laue diffraction pattern to be broken up into monochromatic slices (Fig. 4 ▸), a large proportion of the reflections would be spatially overlapped (Langan *et al.*, 2008 ▸; Coates *et al.*, 2010 ▸). Furthermore, the reflection diffraction intensity is directly related to the volume of the crystal and the volume of the unit cell, making data collection from large unit cells using the low flux available at neutron beams particularly challenging. However, by using protein perdeuteration, which enables data collection from smaller crystals, coupled with high-resolution time-of-flight neutron crystallography, we have been able to collect data for one of the most challenging systems to be studied with neutrons to date.

## Figures and Tables

**Table 1:** Data-collection statistics

Diffraction source	Rigaku MicroMax-007 HF	MaNDi
Wavelength(s) (Å)	1.54	2–4
Temperature (K)	293	293
Detector(s)	R-AXIS IV^++^	40 SNS Anger cameras
Crystal-to-detector distance (mm)	200	450
Rotation range per image (°)	0.25	0
No. of images collected	400	6
Total rotation range (°)	100	120
Exposure time per image	30 s	48 h
Space group	*P*6_1_22	*P*6_1_22
*a*, *b*, *c* (Å)	81.40, 81.40, 242.0	81.31, 81.31, 242.0
α, β, γ (°)	90, 90, 120	90, 90, 120
Resolution range (Å)	19.71–2.35 (2.48–2.35)	14.62–2.30 (2.38–2.30)
Total No. of reflections	228827	43593
No. of unique reflections	20582	16318
Completeness (%)	99.7 (99.8)	74.29 (68.83)
Multiplicity	9.0 (11.0)	2.67 (2.07)
〈*I*/σ(*I*)〉	8.7 (2.70)	5.4 (2.70)
*R*_merge_ (%)	7.6 (27.0)	19.2 (22.8)
*R*_p.i.m._ (%)	2.30 (8.7)	11.1 (15.9)
